# A Buried Sulfonate Molecular Bridge for Synchronous Charge Transport and Defect Passivation in High-Performance Perovskite Solar Cells

**DOI:** 10.1007/s40820-026-02278-6

**Published:** 2026-08-14

**Authors:** Leying Zha, Weilu Ding, Yalin Gao, Xiao Wu, Tinglu Song, Changhua Li, Chenghao Duan, Xinhui Lu, Guilong Cai, Suojiang Zhang

**Affiliations:** 1 Longzihu New Energy Laboratory, Zhengzhou Institute of Emerging Industrial Technology, School of Energy Science and Technology, Henan University, Zhengzhou, 450000 People’s Republic of China; 2 Center of Ionic Liquids and Green Energy, Beijing Key Laboratory of Solid State Battery and Energy Storage Process, Institute of Process Engineering, Chinese Academy of Sciences, Beijing, 100190 People’s Republic of China; 3 State Key Laboratory of Green Chemical Synthesis and Conversion, School of Energy Science and Technology, Henan University, Zhengzhou, 450000 People’s Republic of China; 4 Department of Physics, The Chinese University of Hong Kong, New Territories, Hong Kong, 999077 People’s Republic of China; 5 Experimental Center of Advanced Materials School of Materials Science & Engineering, Beijing Institute of Technology, Beijing, 100081 People’s Republic of China

**Keywords:** Buried interface, Sodium sulfonate salt, Carrier transport, Defect passivation, Perovskite solar cells

## Abstract

The quality of the buried interface between the self-assembled molecules (SAM) and the perovskite layer directly governs the processes of charge carrier transport and non-radiative recombination, which ultimately dictates the efficiency and stability of the inverted perovskite solar cells. However, the simultaneous mitigation of poor SAM layer adhesion and perovskite substrate interface defects remains a significant challenge. Herein, low-cost and readily available 2‑formylbenzenesulfonic acid sodium salt (2‑FAS) is employed as a bifunctional interlayer to molecularly bridge the SAM and perovskite. The benzene ring of 2‑FAS interacts via π–π stacking with the SAM, strengthening adhesion and promoting hole transfer, while its sulfonate group (-SO_3_^−^) coordinates with Pb^2+^ to regulate crystallization and passivate surface defects. As a result, the 2-FAS-modified devices deliver a champion power conversion efficiency of 26.21%, with a significant fill factor of 86.15%. Furthermore, Na^+^ from 2‑FAS occupies A‑site vacancies in the perovskite lattice, which effectively suppresses ion migration and phase transition, thereby enhancing structural integrity. Benefiting from these combined effects, unencapsulated devices retain over 90% of their initial PCE after 4500 h of storage in a nitrogen atmosphere, demonstrating exceptional long-term stability.

**Supplementary Information:**

The online version contains supplementary material available at 10.1007/s40820-026-02278-6.

## Highlights

- Bridging the 2-formylbenzenesulfonic acid salt molecule at the self-assembled molecules/perovskite buried interface enhances charge transfer via π–π stacking and passivates defects via Pb^2+^ coordination, yielding a superior power conversion efficiency of 26.21%.
- The modified devices demonstrate exceptional storage stability, retaining over 90% of their initial power conversion efficiencies after 4500 h in a nitrogen atmosphere without encapsulation.
- Na^+^ from 2-formylbenzenesulfonic acid salt effectively occupy A-site vacancies in the perovskite lattice, reinforcing structural stability and substantially improving device longevity.

**Supplementary Information:**

The online version contains supplementary material available at 10.1007/s40820-026-02278-6.

## Graphical abstract

**Figure:**
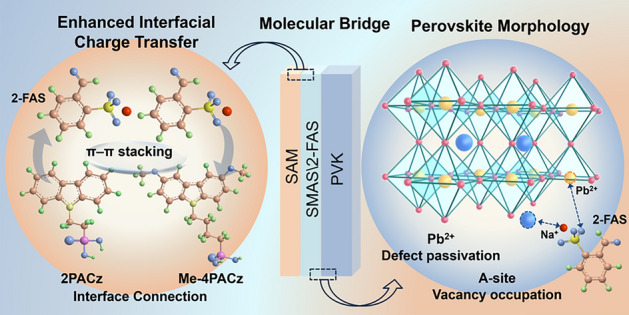

## Introduction

Currently, the perovskite solar cells (PSCs) have emerged as a revolutionary photovoltaic technology, achieving certified power conversion efficiencies (PCEs) exceeding 27% in single-junction devices [1–3]. Among various device configurations, inverted (p-i-n) structured PSCs are particularly promising for commercialization, owing to their compelling advantages of enhanced operational stability, low-temperature processing, and excellent scalability [4, 5]. The performance of inverted PSCs, however, is critically limited by the properties of charge transport layers and their interfaces with the perovskite film. In particular, the hole transport layer (HTL) plays a decisive role in minimizing non‑radiative recombination and enabling efficient hole extraction [6]. To this end, the self-assembled molecules (SAM) of carbazole groups anchored by phosphate groups have become the state‑of‑the‑art HTL material for inverted PSCs. Their widespread adoption stems from a unique combination of advantages: the precise tuning of the substrate work function (WF) and the formation of a favorable interface for charge transfer [7–10].

However, the widespread adoption of SAM-based HTLs is hampered by their structural inhomogeneity. The inherent amphiphilicity of conventional SAM molecules drives detrimental aggregation during deposition, leading to incomplete substrate coverage and a non-compact monolayer [11–13]. These imperfections promote current leakage and disrupt the uniform growth of the overlying perovskite film [14, 15]. Recent strategies to address these issues focus on improving molecular ordering through the incorporation of additives or dispersants into the SAM solution. For instance, Sun et al. improved SAM surface orientation and hole transport by adding the volatile solid additive 1,3,5-trichlorobenzene (TCB) to regulate SAM stacking [16]. Similarly, Xu et al. developed a multi-level strategy to suppress aggregation using the multifunctional solvent additive dibutyl ether (DBtE). The selective electrostatic interaction between DBtE and the PO_3_H_2_ group regulates molecular arrangement and creates a directional steric hindrance effect, thereby minimizing interface defect formation [17]. Nevertheless, residual dispersants or solvents may introduce unintended interface states, specifically undercoordinated Pb^2+^ and A-site vacancies, which trigger severe non-radiative recombination and lattice instability.

A more promising strategy involves inserting a functional interlayer that simultaneously ensures robust physical contact and facilitates efficient electronic coupling between the SAM and perovskite layers [18–21]. For instance, Wolf et al. introduced 1,3-dimethyl-3,4,5,6-tetrahydro-2(1H)-pyrimidinone (DMPU) into the SAM solution to enhance the interfacial interaction and improve wettability, resulting in a more uniform and pinhole-free perovskite film [22]. Ren et al. constructed a p-type heterojunction at the SAM/perovskite interface using the heterocyclic dipolar compounds imidazole hydroiodide (ImHI), which promoted hole extraction and improved the PCE of the device by decreasing the Fermi level offset and energy level mismatch [23]. Zhang et al. demonstrated the ability of a multifunctional π-conjugated molecule with a spirofluorene-bridged backbone to promote robust π–π stacking with SAMs and coordinate with undercoordinated Pb^2+^, thereby passivating interface defects when placed at the SAM/perovskite interface [24]. This approach utilizes functional molecules that robustly adhere to both the SAM and the perovskite, effectively passivate interfacial traps, enhance energy level alignment, and act as a protective barrier against solvent attack or ion migration during perovskite processing [25–28]. However, the rational design of an effective interfacial bridge material necessitates compatibility with adjacent layers, must avoid introducing charge‑transfer barriers or recombination sites, ensure long‑term thermo‑structural stability, and requires a deeper mechanistic understanding of passivation at such multicomponent interfaces.

For this purpose, in this work, the sodium methyl allyl sulfonate (SMAS) and 2-formylbenzenesulfonic acid sodium salt (2-FAS) were selected as buried interfacial bridges, owing to their low cost, facile synthesis, and excellent intrinsic stability. Through this modification, substrate roughness was reduced, and surface free energy was lowered, leading to enhanced wettability and improved uniformity of the subsequently deposited perovskite film. Furthermore, perovskite crystallization was regulated, and interfacial defects were passivated via coordination between the sulfonate groups and Pb^2+^. In contrast to SMAS, the benzene ring in 2‑FAS was utilized to establish π–π stacking with the SAM molecules, thereby facilitating interfacial charge transfer. As a result, a champion PCE of 26.21% was achieved with the 2‑FAS‑based device, outperforming both the control (24.58%) and SMAS‑modified (25.41%) devices. Moreover, 90.06% of the initial efficiency was retained after over 4500 h of storage in nitrogen, demonstrating significantly enhanced long‑term stability. This study presents a targeted interfacial design in which adhesion, defect passivation, and charge transfer are concurrently improved, offering a viable pathway toward efficient and stable perovskite photovoltaics.

## Experimental Section

### Materials

The main materials used in this study are as follows: fluorine-doped tin dioxide glass (FTO, purchased from Suzhou Shangyang Gaichuang Technology Co., Ltd); the SAM colloid solution [2-(9H-Carbazol-9-yl)ethyl]phosphonic acid and [4-(3,6-Dimethyl-9H-carbazol-9-yl)butyl]phosphonic acid (2PACz, Me-4PACz, purchased from Suzhou Liwei New Material Co., Ltd); dimethyl sulfoxide (DMSO, 99.9%), N,N-dimethylformamide (DMF, 99.8%), anisole (As, 99.7%), and ethanol (EtOH, 99.5%) were purchased from Sigma-Aldrich. Lead iodide (PbI_2_, 99.99%) was purchased from TCI. HC(NH_2_)_2_I (FAI, > 99.99%), CH_3_NH_3_I (MAI, 99.9%), and CH_3_NH_3_Cl (MACl, 99.9%) were purchased from Greatcell Solar Materials (Australia). Cesium iodide (CsI, 99.99%) was purchased from Liaoning Preferred New Energy Technology Co., Ltd. Sodium methyl allyl sulfonate (SMAS) was purchased from Macklin Biochemical Co., Ltd. The 2-formylbenzenesulfonic acid sodium salt (2-FAS) was purchased from Aladdin Biochemical Technology Co., Ltd. C_60_ and bathocuproine (BCP) were purchased from Xi’an Yuri Solar Co., Ltd.

### Fabrication of Devices

The FTO (fluorine-doped tin oxide) glasses substrates were ultrasonically cleaned in sequence with detergent, deionized water, acetone, absolute ethanol, and isopropanol (IPA) for 30 min each. The washed substrates were then subjected to UV-ozone treatment for 30 min before use. The self-assembled monolayer (SAM) film was prepared by dissolving 2PACz and Me-4PACz with a 1:3 molar ratio uniformly in ethanol, and the resulting solution was spin-coated onto the FTO substrate at 3000 rpm for 30 s, followed by annealing on a hot plate at 120 °C for 10 min. Then, different masses of SMAS/2-FAS powder were added to anhydrous DMSO solutions. The resulting solutions were spin-coated at 3000 rpm for 30 s and annealed under ambient conditions at 100 °C for 10 min, respectively. For the perovskite precursor, PbI_2_ (1.5 mmol), FAI (1.275 mmol), MAI (0.15 mmol), and CsI (0.075 mmol) were dissolved in 0.9 mL of anhydrous DMF/DMSO (v/v: 4:1) mixed solvent, followed by the addition of 15 mg MACl and 35 mg PbI_2_ to improve the quality of perovskite films. The resulting 1.67 M Cs_0.05_MA_0.1_FA_0.85_PbI_3_ solution was stirred for 6 h as the precursor solution of perovskite. Then, the precursor solution was spin-coated through a two-step program at 1000 rpm for 10 s and 5000 rpm for 35 s in the glove box. At 15 s before the end of the program, approximately 150 µL of the anti-solvent (As) was rapidly dropped on the center of the substrates and heated at 120 °C for 10 min to form a dense perovskite film. After cooling to room temperature, a 25 nm electron transport layer of C_60_ was deposited on the top of the perovskite film using a high-temperature evaporation instrument, followed by the evaporation of a 7 nm BCP layer. Finally, an Ag layer with a thickness of 100 nm was used through thermal evaporation as the back contact electrode.

### Device Characteristic

The illuminated *J-V* characteristics of devices were measured on a programmable source meter (B2901BL, Enlitech) under 1 sun AM 1.5G (100 mW cm^−2^) solar illumination with areas of 0.0214 cm^2^ (SS-X50, Enlitech), calibrated by the certified standard silicon solar cell (SRC-2020, Enli Technology Co., Ltd.). The External quantum efficiency (EQE) spectra of devices were recorded through the solar cell spectral response measurement system (QE-R, Enlitech). The space charge limited current (SCLC) curve of the single-carrier device was recorded in the dark using the Keithley 2400 source table. EIS was obtained at 1.09 V bias under dark conditions on a CHI660E electrochemical workstation. The electrical conductivity measurement was evaluated by devices with structures of FTO/C_60_/BCP/Ag, FTO/SMAS/C_60_/BCP/Ag, and FTO/2-FAS/C_60_/BCP/Ag. Current density–voltage (*J-V*) characteristics were measured using a source meter unit in a two-probe configuration. The voltage was swept from + 3 to −3 V with a fine step size of 0.02 V and a delay time of 10 ms at each measurement point to ensure steady-state current recording. All measurements were performed in the dark and within a nitrogen atmosphere. For the light stability test, the devices were subjected to continuous illumination under LED. The tests were conducted in a nitrogen-filled glovebox with oxygen and water levels kept below 0.1 ppm, at room temperature (25 °C).

## Results and Discussion

### Interface Modification of SAMs

We introduced sodium methylallyl sulfonate (SMAS) and 2-formylbenzenesulfonic acid sodium salt (2-FAS) at the HTL/perovskite interface to improve the buried interface quality and enhance device performance. As shown in Fig. S1, the potential interaction mechanisms between SMAS or 2-FAS and the interface were preliminarily explored by analyzing their chemical structure and electrostatic potential (ESP) distributions. The sulfonic acid group (-SO_3_^−^) in the ESP diagrams of SMAS and 2-FAS exhibits lower electrostatic potential regions (red), suggesting a strong binding affinity toward uncoordinated Pb^2+^ in the perovskite lattice, therefore reducing non-radiative recombination centers at the buried interface [29]. Further, in contrast with SMAS, 2-FAS incorporates a benzene ring moiety, which can form π–π conjugative interactions with SAM molecules on the HTL surface. This unique structural feature is anticipated to facilitate interfacial charge-transfer dynamics.

**Fig. 1:**
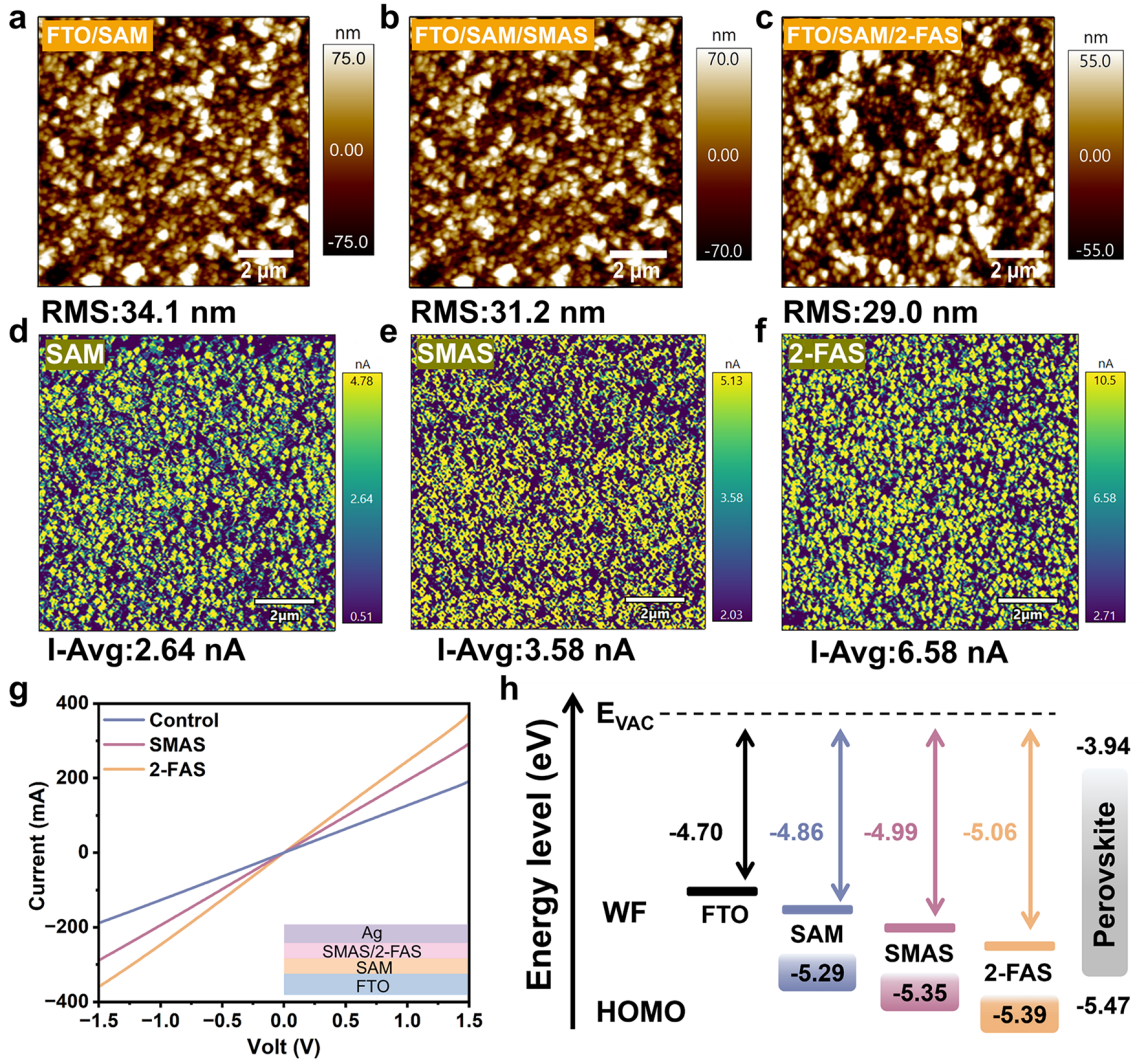
Morphology and properties of the buried interfaces. **a**–**c** AFM images and **d**–**f** C-AFM images of SAM, SAM/SMAS, and SAM/2-FAS films at the nanometer scale. **g** *I-V* curves for the pure HTL device without and with SMAS and 2-FAS. **h** Energy level diagram for SAM, SMAS, and 2-FAS films

To elucidate the morphological influence of SMAS and 2-FAS on SAM substrates, we detected the surface roughness of different films using atomic force microscopy (AFM) [30]. The pristine SAM/FTO film exhibits a relatively high root-mean-square (RMS) roughness of 34.1 nm, which is attributed to the FTO surface roughness (38.6 nm) and the intrinsic aggregation tendency of SAM molecules, resulting in non-uniform coverage of the underlying substrate (Figs. S2–S4 and 1a). After the SMAS and 2-FAS deposition, the RMS roughness of the SAM substrates decreased to 31.2 and 29.0 nm, respectively (Fig. 1b, c). A smoother and more uniform buried interface is critical for improving the spreading uniformity of the perovskite precursor solution atop the HTL, which in turn favors the fabrication of high-quality perovskite film with reduced defect density. Contact angle measurements further confirmed the influence of the SMAS and 2-FAS coatings on the SAM surface wettability. Compared with SAM film (38.76°), the contact angles of perovskite precursor solution with SAM/SMAS and SAM/2-FAS films decreased to 24.03° and 17.00°, respectively (Fig. S5a–c). This improved wettability accelerates the diffusion of the perovskite precursor solution, therefore promoting perovskite crystallization [31]. And the water contact angle shown in Fig. S5d–f changed from 100.71° (SAM) to 84.62° (SMAS) and 77.52° (2-FAS), which indicated that the hydrophilicity of the film surface has increased. The films exhibit similar wetting tendencies toward water and precursor solution, which is the strong evidence of the specific changes that occur on the surface after the introduction of the interface layer.

Additionally, to elucidate the effects of interface modification on charge transport properties, conductive atomic force microscopy (C-AFM) measurements were performed to evaluate the conductivity variations of the interface films [32]. By quantifying the local surface current values, the interfacial charge-transfer capability can be directly visualized. As demonstrated in Fig. 1d–f, the average surface current of SAM substrates is notably increased after SMAS and 2-FAS modification, indicating a more effective interfacial charge transport. Meanwhile, the current–voltage (*I-V*) characteristic of the device is further tested, and the structure of the test sample is depicted in the inset of Fig. 1g. It can be clearly seen that the introduction of SMAS and 2-FAS interlayers results in a steeper curve slope, a parameter directly correlated with film conductivity. The calculated electrical conductivity (*σ*) and hole mobility (*μ*_hole_) were significantly higher in SMAS- and 2-FAS-treated films, with the 2-FAS sample exhibiting the greatest enhancement (Table S1) [33, 34]. This improvement of hole mobility is directly associated with the passivation of vacancy defects and the formation of π–π conjugative interactions between the benzene ring of 2-FAS and the SAM layer, which further suppresses charge accumulation at the interface.

Furthermore, ultraviolet–visible (UV–Vis) absorption spectroscopy (Fig. S7) revealed that neither SMAS nor 2-FAS introduction induces additional optical loss, ensuring unimpeded light absorption by the perovskite layer. UV-photoelectron spectroscopy (UPS) was employed to determine the energy band alignment of the modified films [35, 36]. The UPS spectra are displayed in Fig. S8, with the corresponding quantitative data summarized in Table S2. Based on the secondary electron valence band edge (E_onset_) and cutoff region (E_cutoff_) of the UPS spectra, we acquired the maximum valence band (VBM) values of the films. The energy level graph is presented in Fig. 1h, the WF of SAM/SMAS and SAM/2-FAS films was substantially reduced, leading to better energy level alignment with the perovskite VBM [29]. This optimized energy level matching helps lower the hole extraction barrier and suppresses non-radiative recombination at the SAM/perovskite interface, thereby improving charge transfer and device performance.

### Perovskite Morphology Regulation

**Fig. 2:**
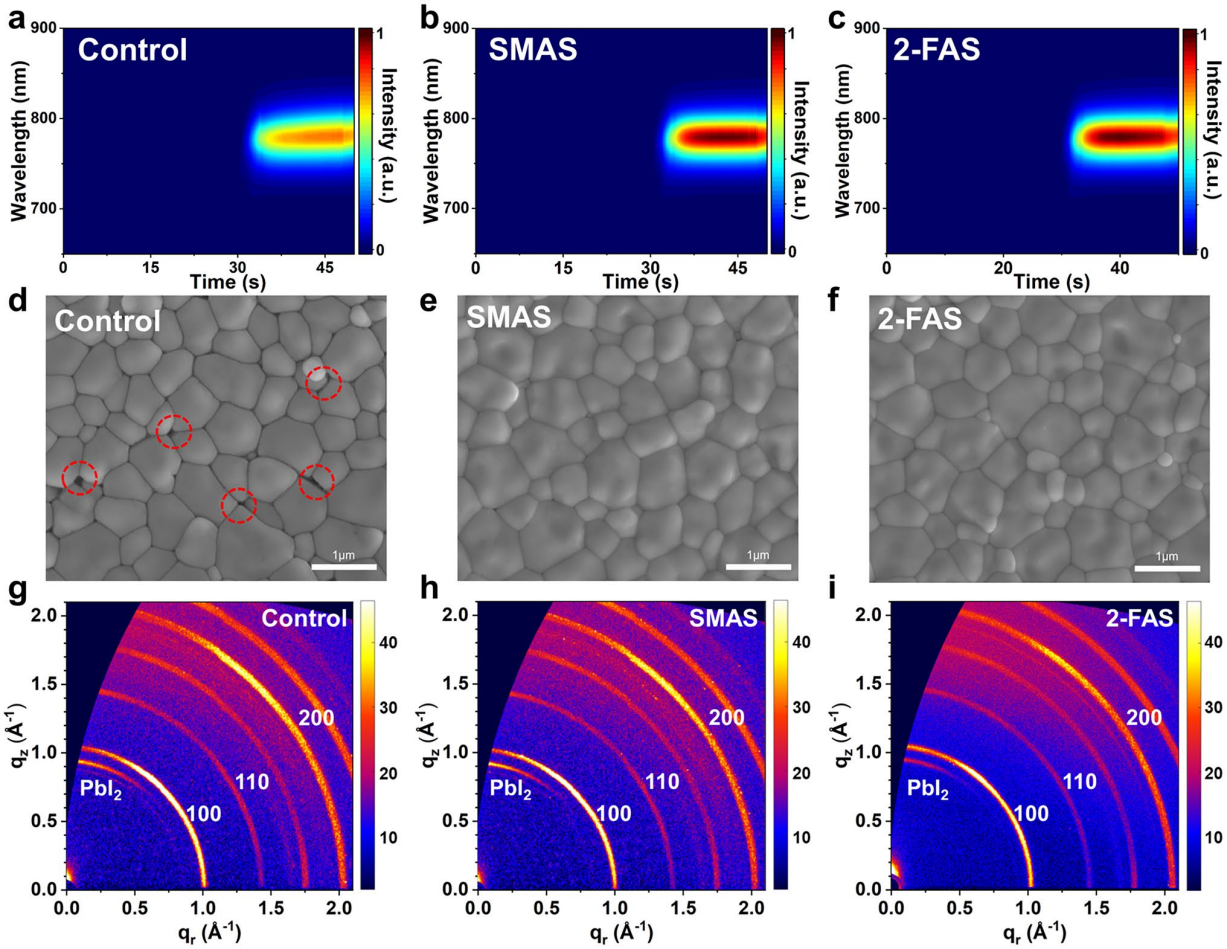
Perovskite morphology characterizations. In situ PL during spin-coating of **a** SAM, **b** SMAS, and** c** 2-FAS-based perovskite films. SEM images of **d** SAM, **e** SMAS, and** f** 2-FAS-based bottom interface perovskite films. GIWAXS images of** g** SAM, **h** SMAS, and **i** 2-FAS-based perovskite films

To investigate the effect of the buried interface treatment on the film-forming quality of the upper perovskite layer, the in situ photoluminescence (PL) measurement during the perovskite spin-coating was first conducted [37]. The steady-state PL intensity contour maps for the perovskite films fabricated on the control, SMAS-treated, and 2-FAS-treated substrates are presented in Fig. 2a–c. Following the anti-solvent quenching during spin-coating, a distinct and immediate enhancement in PL intensity is observed for the 2-FAS samples relative to the control sample (Fig. S9). This demonstrates that adding SMAS and 2-FAS intermediate layers is beneficial for perovskite crystallization, thereby reducing buried interface defect states and suppressing non-radiative recombination. Consequently, the resulting high-quality perovskite film is directly linked to an improved open-circuit voltage (*V*_OC_) of the device. To further visually investigate the influence of the buried interface on perovskite film morphology, the bottom surface of the perovskite films was observed by scanning electron microscopy (SEM) illustrated in Fig. 2d–f. The perovskite film deposited directly on the SAM substrate exhibits a defective morphology characterized by scattered voids and wide grain boundaries (Fig. 2d), which leads to severe non-radiative recombination. In contrast, as clearly shown in Fig. 2e, f, the bottom interface of the perovskite films deposited on the modified SAM substrates exhibits a dense crystal morphology, especially the larger-sized and more densely perovskite crystals formed on the 2-FAS-modified substrate [38]. We calculated the grain size of perovskite on different buried interfaces through SEM images of the bottom interface perovskite films. As shown in Fig. S10, a significant increase in average grain size was measured, rising from 559 nm for the control film to 764 nm for the SMAS-treated film and 815 nm for the 2-FAS-treated film. Furthermore, cross-sectional SEM images shown in Fig. S11 reveal that the perovskite deposited on the SAM layer contains voids on the bottom surface and poorer morphology orientation [39]. Compared with the control sample, the SMAS- and 2-FAS interface-modified samples exhibit uniform and vertically aligned perovskite crystal growth without visible pores. Such vertically oriented and large-grained perovskite can reduce defect states at grain boundaries, facilitating the transfer of free charges and reducing non-radiative recombination losses.

To further understand the influence of different buried interfaces on perovskite crystallization, X-ray diffraction (XRD) and grazing-incidence wide-angle X-ray scattering (GIWAXS) characterization were performed [40]. The XRD pattern of the perovskite film deposited on the SAM substrate exhibits distinct characteristic peaks at 14.14°, 20.01°, and 28.33°, corresponding to the peak of PbI_2_ and the (100), (110), and (200) crystal planes of the perovskite crystal, respectively (Fig. S12a) [41]. In comparison, the characteristic peak intensity of PbI_2_ in perovskite films deposited on SMAS and 2-FAS substrates decreased (Fig. S12b), indicating that optimizing the buried interface can improve the crystallization process of perovskite films. To further validate this conclusion, we conducted GIWAXS measurements on different perovskite films at an incident angle of 0.1° in Fig. 2g–i [42, 43]. It can be observed in 2D GIWAXS spectra that the perovskite crystal structures deposited on different buried interfaces exhibit similar phase compositions, while the intensity of the PbI_2_ diffraction ring in the 2-FAS sample significantly decreased. Moreover, according to the linecuts (Fig. S13) extracted from the 2D GIWAXS pattern, the diffraction ring of the PbI_2_ phase (q = 0.91 Å^−1^) is decreased in the 2-FAS sample, which is consistent with the XRD result.

Moreover, to evaluate the impact of the buried layer on residual stress in perovskite film, we performed the grazing-incidence X-ray diffraction (GIXRD) characterizations (Fig. S14) [44, 45]. As the inclination angle *ψ* increases from 0.3° to 1.8°, the scattering peak of the control perovskite gradually shifts toward lower angles, implying the presence of tensile stress. Under identical testing conditions, the diffraction peak shift angle of perovskite films deposited on SMAS and 2-FAS substrates was significantly reduced. The residual stress of the perovskite films was quantified using the Bragg formula, and the slope of the fitted line represents the magnitude of residual strain. As shown in Fig. S15, the reduction of slope proves that optimizing the buried interface can reduce the residual tensile stress of perovskite films. This stress reduction is attributed to the interaction between the -SO_3_^−^ groups of SMAS or 2-FAS and the perovskite precursors, which regulates the crystallization process to reduce the formation of crystal defects and release the residual stress inside the perovskite. Additionally, UV–vis absorption spectra show a slight enhancement in absorption for perovskite films on SMAS and 2-FAS substrates presented in Fig. S16a, which is directly correlated with the improved crystallinity and reduced defect density of the perovskite film. The optical band gap (E_g_) values remain unchanged at 1.54 eV for all perovskite films measured by UV–vis absorption spectroscopy (Fig. S16b).

### Interaction Mechanism

**Fig. 3:**
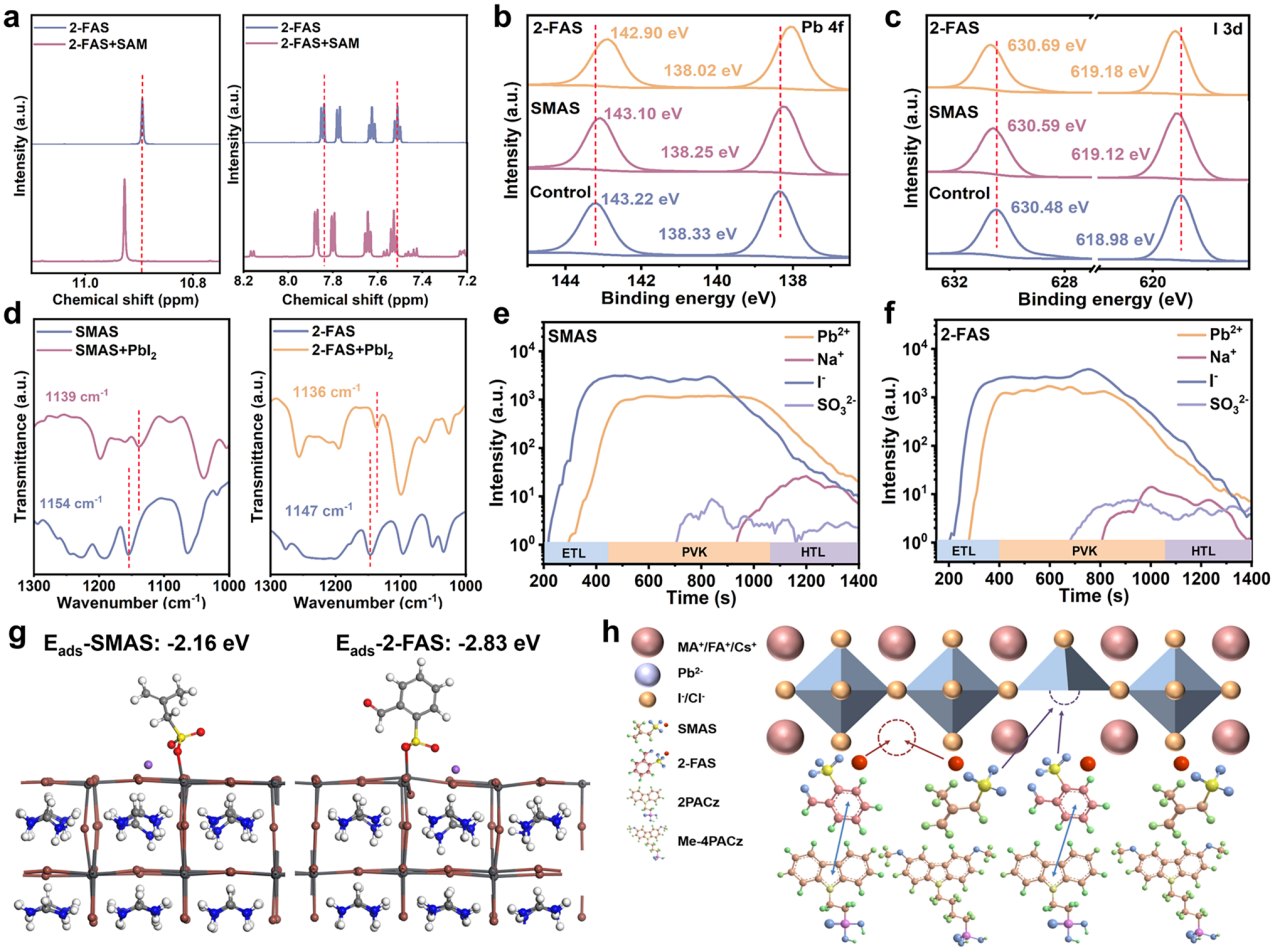
Interaction characterization and mechanism calculation. **a** ^1^H nuclear magnetic resonance spectra of 2-FAS and 2-FAS + SAM in DMSO-d6 solution. High-resolution XPS spectra of **b** Pb 4*f* and **c** I 3*d* for SAM, SMAS, and 2-FAS-based perovskite films. **d** FTIR spectra of SMAS (or 2-FAS) and SMAS (or 2-FAS) + PbI_2_. TOF–SIMS depth profiles of **e** SMAS and **f** 2-FAS-based devices. **g** Proposed adsorption configuration of SMAS and 2-FAS molecule on perovskite layer through DFT calculations. **h** Schematic illustration of the interaction between the molecular layer and its neighboring layers

To elucidate the interaction mechanisms between SMAS and 2-FAS with the SAM layer, we investigated ^1^H nuclear magnetic resonance (NMR) measurements on different samples using DMSO-d6 solution as the solvent. As shown in Fig. 3a, the ^1^H NMR spectrum of the 2-FAS:SAM mixture exhibits a downfield chemical shift belonging to the –CHO and the olefin double bond on the benzene ring of 2-FAS, indicating the π–π stacking interactions between 2-FAS and the SAM. On the contrary, no significant chemical shift was observed in the SMAS:SAM sample (Fig. S17), suggesting the absence of strong intermolecular interactions between SMAS and SAM. The interaction between SMAS and 2-FAS with perovskite was further investigated through X-ray photoelectron spectroscopy (XPS) and Fourier-transform infrared spectroscopy (FTIR) measurements.

Figure 3b displays the high-resolution Pb 4*f* XPS spectra of the perovskite bottom interfaces under different modification conditions. The control perovskite sample shows two characteristic peaks at 143.22 and 138.33 eV, which correspond to Pb 4*f*_5/2_ and Pb 4*f*_7/2_, respectively. With SMAS or 2-FAS modification, these Pb 4*f* peaks shift toward lower binding energy (143.10 and 138.25 eV for SMAS, 142.90 and 138.02 eV for 2-FAS), suggesting that the electron cloud density near Pb increases. This phenomenon conforms to the typical characteristics of Lewis acid–base coordination, where the -SO_3_^−^ group in SMAS and 2-FAS acts as a Lewis base to donate electron pairs, forming coordination with the Lewis acid Pb^2+^ in the perovskite. Thereby, the -SO_3_^−^ can coordinate with uncoordinated Pb^2+^ at the bottom interface of perovskite film, effectively passivating defect states [46, 47]. Meanwhile, compared with the control sample, the XPS spectra of the I 3*d* peak shift toward the higher binding energy after depositing the SMAS and 2-FAS layer, which is due to the metal cation Na^+^ occupying A-site defects in the perovskite crystal (Figs. 3c and S18, S19). The incorporation of Na^+^ into A-site vacancies modulates the local electronic environment of I^−^, reducing their electron density and thus increasing the I 3*d* binding energy. In addition, the interactions between SMAS and 2-FAS with perovskite films were also analyzed with FTIR spectra. As depicted in Fig. 3d, the characteristic peak at around 1154 cm^−1^ in the FTIR spectrum of pure SMAS is designated as the S=O stretching vibration. Upon mixing SMAS with PbI_2_, this peak shifts to a lower wavenumber (1139 cm^−1^). This displacement indicates the coordination between -SO_3_^−^ in SMAS and Pb^2+^ in the perovskite precursor, which is assertive evidence of the coordination of the sulfonic acid group to the Lewis acid metal center (Pb^2+^). A similar shift of the S=O stretching vibration peak is also observed in the 2-FAS:PbI_2_ sample, confirming analogous coordination behavior [48].

The spatial distribution of SMAS and 2-FAS molecules at the SAM/perovskite interface was further verified by time-of-flight secondary ion mass spectrometry (TOF–SIMS) technology, as shown in Fig. 3e, f [49, 50]. The signals for Na^+^ and -SO_3_^−^ in SMAS and 2-FAS molecules are localized predominantly at the bottom interface of perovskite, which is because Na^+^ occupies A-site vacancies while -SO_3_^−^ coordinates with uncoordinated Pb^2+^ in perovskite crystals. Density functional theory (DFT) calculations were performed to quantitatively elucidate the relationship between molecular structure and interfacial interaction strength [51]. First, the adsorption energy (E_ads_) of the -SO_3_^−^ group with the Pb^2+^ vacancy site of perovskite was calculated. As displayed in Fig. 3g, the E_ads_ of SMAS on the Pb^2+^ vacancy is 2.16 eV, but higher for the 2-FAS (2.83 eV). The formation energy of the Na^+^ occupying the A-site vacancy was further calculated as 0.38 and 0.41 eV for SMAS- and 2-FAS-modified perovskite, respectively (Figs. S20 and S21), suggesting that Na⁺ from both SMAS and 2-FAS can easily occupy A-site vacancies. Particularly, the π–π interaction force between the 2-FAS and SAMs was calculated to be about 2.19 eV, confirming the stability of this π–π stacking interaction (Fig. S22). Based on these results, the interfacial interaction mechanism of the SAM/perovskite modified by SMAS or 2-FAS is schematically illustrated in Fig. 3h. Both SMAS and 2-FAS are uniformly deposited on the SAM layer, covering the FTO substrate that is not covered by SAM molecules. Meanwhile, the -SO_3_^−^ groups in SMAS and 2-FAS molecules regulate the crystallization process of perovskite by coordinating with Pb^2+^ in the precursor, inducing the formation of a more ordered perovskite crystal structure and reducing the formation of defect states. The Na^+^ cations derived from SMAS and 2-FAS occupy the A-site vacancies in the perovskite lattice to stabilize the crystal structure of perovskite and improve the stability of the corresponding devices. Uniquely, 2-FAS engages in π–π interaction with the SAM molecules to construct a charge-transfer bridge, which facilitates the extraction of free charges.

### Device Performance

**Fig. 4:**
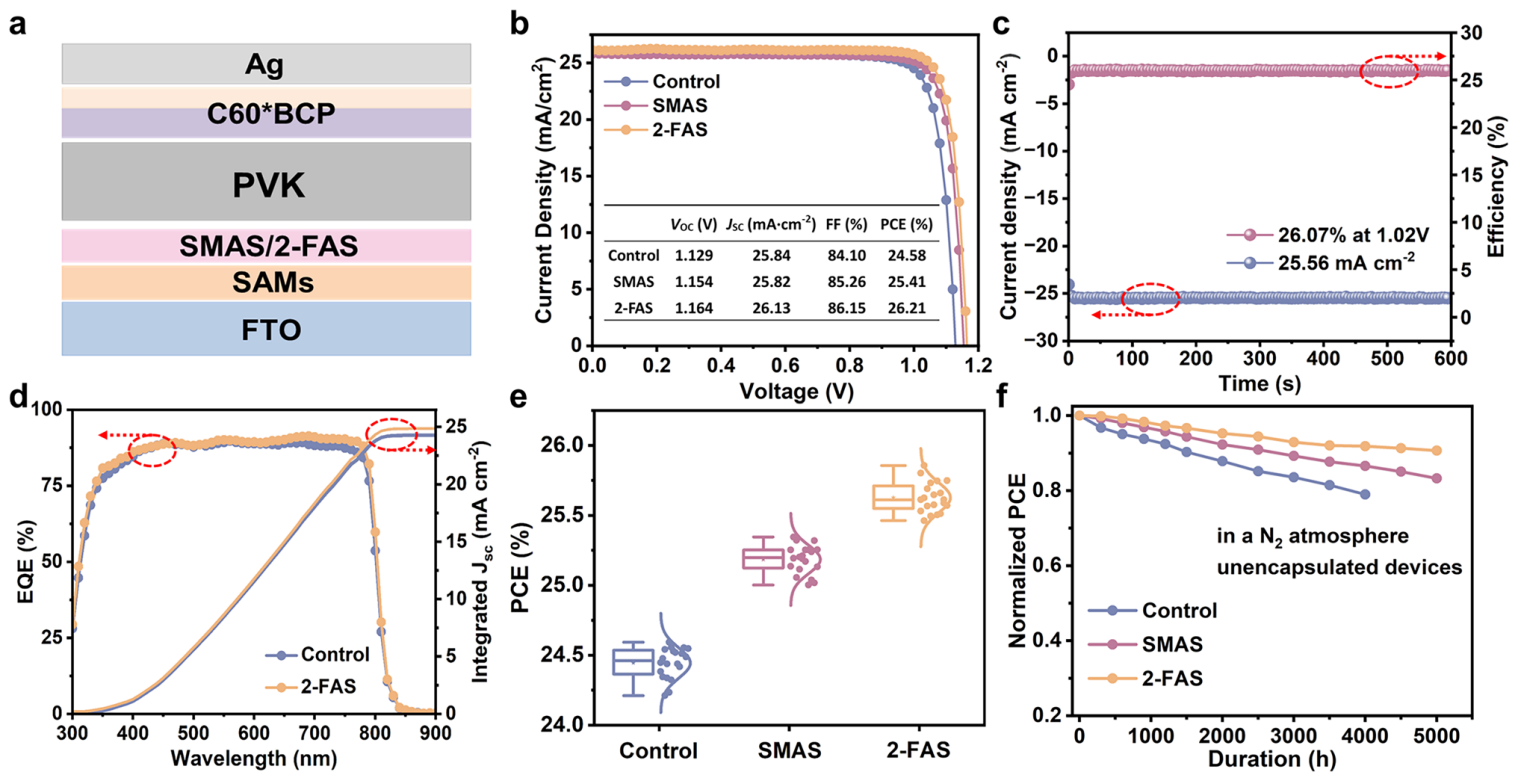
Photoelectric performance and stability of the devices. **a** Schematic illustration of the device architecture incorporating the buried layer. **b** *J–V* curves of the control, SMAS-, and 2-FAS-treated p-i-n devices. **c** Stabilized efficiency output at the maximum power point of the champion devices under AM 1.5 G illumination. **d** EQE spectra alongside the corresponding integrated *J*_SC_ for the control and 2-FAS-treated devices. **e** Statistical distribution of PCE for devices based on SAM, SAM/SMAS, and SAM/2-FAS films. **f** Stability of unencapsulated devices without and with SMAS or 2-FAS stored in a nitrogen atmosphere

To evaluate the impact of various buried interfaces on the photoelectric performance of PSCs, we fabricated PSCs with the structure of FTO/SAM/(SMAS or 2-FAS)/perovskite/C₆₀/BCP/Ag (Fig. 4a). Firstly, SMAS and 2-FAS solutions with gradient concentrations (1.0, 1.5, 2.0, and 2.5 mg mL^−1^) were deposited on the SAM layer to prepare corresponding devices. The current density–voltage (*J-V*) curves of these devices measured under reverse scanning with standard simulated AM 1.5G light are shown in Fig. S23, with the associated photovoltaic parameters summarized in Tables S3 and S4. Apparently, the optimal device performance was achieved at a concentration of 2.0 mg mL^−1^ for both modifiers. Considering the functional groups at different positions on the benzene ring influence the final interaction outcomes, we examined the impact of molecular structure variations on device performance. Figure S24 shows the electrostatic potentials and the direction of dipole moment at the ortho, meta, and para positions of the 2-FAS molecule. Notably, the vertical component of the molecular dipole moment (μ⊥) at the ortho position is significantly larger than that at the meta and para positions. However, it should be noted that the Gaussian calculations presented here can only provide qualitative or approximate evidence. During the spin-coating process, variations in the retained volumes of different molecular configurations may lead to differences in the packing density of the film.

In addition, the photoelectric conversion efficiency of PSCs containing these different 2-FAS isomers was tested in Fig. S25. The device based on ortho-2-FAS exhibits substantially higher efficiency than its meta- and para-substituted counterparts. Thus, by combining the electrostatic calculation with the experimental efficiency data, we selected ortho-2-FAS as the representative molecule to further investigate the influence of different interface treatment molecules (SMAS and 2-FAS) on SAM/perovskite interactions and their subsequent effects on device performance. To further investigate the role of the interface, we measured the *J-V* curves of devices based on various substrate configurations (Fig. S26 and Table S5). It can be found that devices employing only SMAS (15.57%) or only 2-FAS (17.87%) on the substrate yielded much lower efficiencies than those with the SAM/SMAS and SAM/2-FAS architectures. This poor performance confirms that SMAS or 2-FAS alone are insufficient as hole-selective layers. The significant efficiency improvement observed upon their deposition onto the SAM layer highlights their critical function as interfacial modifiers between the SAM and the perovskite.

In light of the aforementioned results and a holistic evaluation of all influencing factors, we conducted a comparative analysis of the optimal PCE of devices based on different buried interfaces (see the *J-V* curves in Fig. 4b and photovoltaic parameters inset). The control device exhibited a PCE of 24.58%, with an open-circuit voltage (*V*_OC_) of 1.129 V, a current density (*J*_SC_) of 25.84 mA cm^−2^, and a fill factor (FF) of 84.10%. The SMAS-modified device achieved an efficiency of 25.41%, while the 2-FAS-treated device demonstrated superior performance with a PCE of 26.21%, along with a *V*_OC_ of 1.164 V, a *J*_SC_ of 26.21 mA cm^−2^, and an FF of 86.15%. The enhancements in *V*_OC_ and FF of the 2-FAS-modified devices are attributed to the optimization of energy level structure and passivation of perovskite defects by 2-FAS, which collectively mitigate non-radiative recombination losses at the SAM/perovskite interface. To confirm the reliability of the photovoltaic performance, we tested the steady-state output power and current at the maximum voltage of a 2-FAS-based device; the device yielded a stabilized efficiency of 26.07% and a photocurrent density of 25.56 mA cm^−2^, which is consistent with the *J-V* test results (Fig. 4c) [52]. External quantum efficiency (EQE) measurements were further performed to elucidate the enhancement mechanism of the 2-FAS-modified interface. The integrated photocurrent density derived from the EQE spectrum showed that the 2-FAS-modified PSCs exhibited an integrated *J*_SC_ of 24.92 mA cm^−2^, which is higher than that of the control device (Fig. 4d) [46].

To verify the reliability and superiority of the 2-FAS modification strategy, we conducted statistical analysis on the photovoltaic parameters of multiple devices with different interface configurations. As shown in Figs. 4e and S27, the 2-FAS-modified devices exhibited excellent reproducibility and consistent performance superiority, indicating the robustness of the modification strategy. Device stability is one of the most significant factors to evaluate the overall performance of PSCs due to the significant environmental sensitivity of perovskite [53]. To evaluate the long-term stability of the devices, we systematically monitored the storage stability and light-soaking stability of the unencapsulated PSCs under a nitrogen environment [54]. After 4500 h of storage, the 2-FAS-modified devices retained 90.06% of their initial PCE, outperforming the SMAS-based (83.27%) and control (78.19%) devices, as illustrated in Fig. 4f.

Moreover, the stability of the unencapsulated devices under various environmental conditions was also evaluated. The PCE of the PSCs was periodically monitored while they were exposed under constant illumination with LED light in a nitrogen atmosphere (Fig. S28a). After 1800 h, the devices treated with 2-FAS on average maintained around 90.27% of their initial PCE, but the control devices fell below 80% after 1500 h. Furthermore, the thermal stability of the devices was assessed under 65 °C heating in a N_2_ atmosphere. Compared to the control device (67.04%), the 2-FAS-modified devices maintained 89.02% of their initial efficiency after 1000 h (Fig. S28b). Moreover, after being stored in ambient air (15−25% RH) for 900 h, the unencapsulated 2-FAS device can still maintain 90% of its initial efficiency (Fig. S28c). Finally, we compared the operational stability of the unencapsulated devices under continuous MPP tracking (Fig. S29). The 2-FAS‑based device retained 83.7% of its initial PCE for 1000 h, outperformed the control device, which dropped to 63.8% in 550 h. The fundamental reason for the improved stability is that the optimized buried interface constructed by 2-FAS not only stabilizes the crystal structure of the perovskite film but also inhibits the formation and evolution of defect states in the perovskite material, thereby enhancing the environmental tolerance of the devices.

### Device Physical

**Fig. 5:**
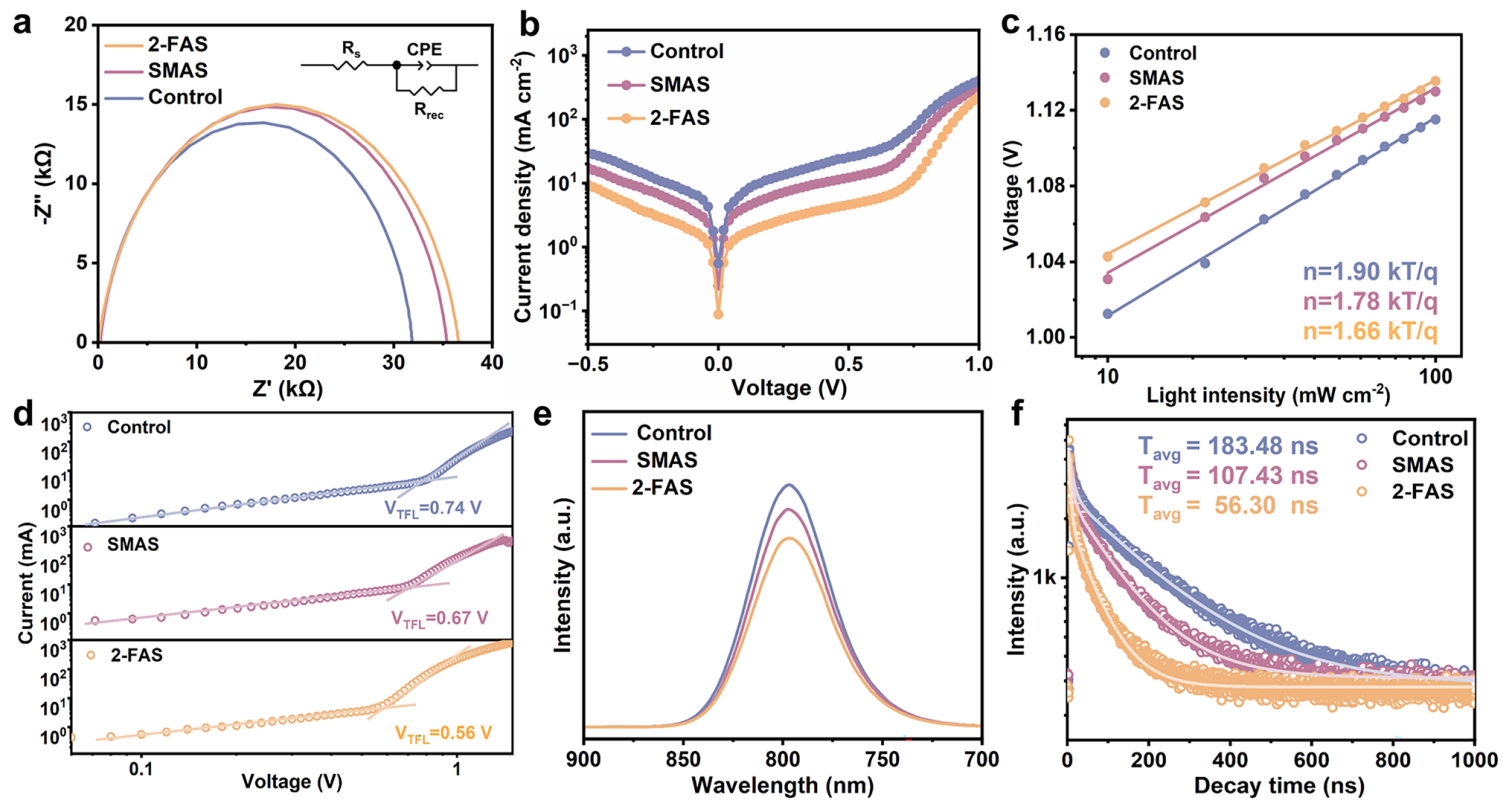
Device physical characterizations. **a** Fitted spectra of Nyquist plots under dark conditions. **b** *J-V* characteristics measured in the dark for devices fabricated without and with SMAS or 2-FAS interlayers. **c** Plots illustrating the dependence of voltage on light intensity for devices incorporating SAM, SMAS, and 2-FAS films. **d** SCLC measurement for the hole-only devices based on SAM, SAM/SMAS, and SAM/2-FAS under dark conditions. **e** PL and **f** TRPL spectra of perovskite layers on FTO/SAM (without and with SMAS or 2-FAS layer)

Moreover, electrochemical impedance spectroscopy (EIS) was used to investigate charge transport and recombination kinetics of the PSCs [55]. The Nyquist plots presented in Fig. 5a were fitted with an equivalent circuit model that consists of a charge recombination resistance (R_rec_) and a series resistance (R_s_). Devices modified with SMAS and 2-FAS exhibited an obviously higher R_rec_ in the low-frequency region compared to that of the control device, indicating a suppression of charge recombination at the interface, thereby substantially improving the charge-transfer capability of the device. Subsequently, dark *J-V* curves of the devices depicted in Fig. 5b revealed that the reverse saturation current density of devices based on SMAS and 2-FAS decreased, implying that the interface defects were effectively reduced, hence mitigating the photocurrent leakage. To quantitatively evaluate the suppression of non-radiative recombination, we measured the electroluminescence quantum efficiency (EQE_EL_) of the devices [56]. The non-radiative recombination-induced *V*_OC_ loss (Δ*V*_OC, nonrad_) was determined to be 111 mV for the control device and 89 mV for the 2-FAS-treated device (Fig. S30). The lowest Δ*V*_OC, nonrad_ value observed for the 2-FAS-treated device explains its higher *V*_OC_ and improved overall device performance, underscoring the effectiveness of buried interface modification.

Another aspect to consider is the impact of the buried interface on the carrier recombination mechanisms in PSCs. The *V*_OC_ of different devices was measured under various light intensities [57]. The ideality factors (n), derived from linear fitting based on Formula (S5) (see the supporting information), decreased from 1.90 kT q^−1^ for the control device to 1.78 kT q^−1^ and 1.66 kT q^−1^ for the SMAS- and 2-FAS-modified devices, respectively (Fig. 5c). A smaller value of n signifies fewer defects and trap-assisted carrier recombination in PSC. In addition, the space-charge-limited current test (SCLC) measurement was utilized to quantify the trap-state in perovskites to better comprehend the effect of the buried interface on perovskite defect states [45, 58]. The defect density (N_trap_) in the perovskite films was calculated from the SCLC curves of the hole-only devices (Fig. 5d) with FTO/SAMs/PVK/(SMAS/2-FAS)/MoO_3_/Ag structures using Formula (S6). The V_TFL_ values of the control, SMAS/PVK, and 2-FAS/PVK-based devices are 0.74, 0.67, and 0.56 V, while the corresponding N_trap_ values are 2.71 × 10^15^, 2.45 × 10^15^, and 2.05 × 10^15^ cm^−3^, respectively. These results confirm that the modified interfaces effectively reduce defect concentrations in the perovskite layer.

Moreover, steady-state photoluminescence (PL) and time-resolved PL (TRPL) spectroscopy were employed to study carrier transport dynamics in perovskite films [59, 60]. As shown in Fig. 5e, the PL intensity of the perovskite films was significantly decreased following the introduction of the SMAS and 2-FAS interfacial layer, especially for the films modified with 2-FAS, confirming that the photo-generated carriers in the perovskite film were rapidly extracted. This PL quenching phenomenon indicates a significant enhancement in carrier injection efficiency, which is attributed to the bridging effect of 2-FAS between the SAM and the perovskite. As shown in Fig. 5f, the TRPL spectrum was fitted with a double-exponential decay equation, and the relevant carrier lifetimes were summarized in Table S7[61]. With the introduction of SMAS and 2-FAS interface layers, the slow decay lifetime (τ_2_) of the perovskite film decreased from 193.58 ns to 116.22 and 62.60 ns, while the fast decay lifetime (τ_1_) decreased from 10.89 ns to 8.12 and 4.77 ns. Additionally, compared with the control sample (183.48 ns), the average lifetime (τ_avg_) of the SMAS and 2-FAS samples decreased to 107.43 and 56.30 ns, respectively. This means that the optimized buried interface reduces the non-radiative recombination rate at the SAM/perovskite interface by enhancing the extraction and transport of photo-generated charge carriers.

## Conclusion

In summary, this work establishes that molecular engineering of the buried SAM/perovskite interface with sulfonate-based modifiers significantly enhances interfacial integrity and device performance. Combined theoretical and experimental analyses confirm that both SMAS and 2‑FAS enhance substrate planarity and favor interfacial energy alignment. Their sulfonate groups further coordinate with Pb^2+^ to regulate perovskite crystallization and concurrently passivate defect states. Significantly, the benzene ring in 2‑FAS establishes stable π–π stacking with the SAM, and the interaction energy calculated is 2.19 eV. Therefore, in contrast to SMAS, 2‑FAS creates a conjugated bridge across the interface and thereby facilitates efficient charge transfer. Consequently, the 2‑FAS‑modified inverted PSCs deliver a champion efficiency of 26.21% along with a high fill factor of 86.15%, clearly surpassing the control (24.58%) and SMAS‑based devices (25.41%). Furthermore, these devices exhibit outstanding operational stability, retaining 90.06% of their initial efficiency after 4500 h in nitrogen. This work thus demonstrates a rational interfacial design strategy that concurrently addresses efficiency and stability, offering a generalizable pathway toward high‑performance perovskite photovoltaics.

## Supplementary Information

Below is the link to the electronic supplementary material.

Supplementary file1 (DOCX 26194 kb)
