# Supplementary material for: A Buried Sulfonate Molecular Bridge for Synchronous Charge Transport and Defect Passivation in High-Performance Perovskite Solar Cells
Source: Nanomicro Lett. 2026 Aug 14;19:35. doi: 10.1007/s40820-026-02278-6 (PMC13476400; doi:10.1007/s40820-026-02278-6)
Supplement: Supplementary file 1 [file 40820_2026_2278_MOESM1_ESM.docx]

Supporting Information for

**A Buried Sulfonate Molecular Bridge for Synchronous Charge Transport and** **Defect Passivation in High-Performance Perovskite Solar Cells**

Leying Zha,^1^ Weilu Ding,^2^ Yalin Gao,^1,3^ Xiao Wu,^4^ Tinglu Song,^5^ Changhua Li,^1^ Chenghao Duan,^1,3*^ Xinhui Lu,^4^ Guilong Cai,^1,2,3*^ Suojiang Zhang,^1,2,3*^

^1^ Longzihu New Energy Laboratory, Zhengzhou Institute of Emerging Industrial Technology, School of Energy Science and Technology, Henan University, Zhengzhou 450000, P. R. China

^2^ Center of Ionic Liquids and Green Energy, Beijing Key Laboratory of Solid State Battery and Energy Storage Process, Institute of Process Engineering, Chinese Academy of Sciences, Beijing 100190, P. R. China

^3^ State Key Laboratory of Green Chemical Synthesis and Conversion, School of Energy Science and Technology, Henan University, Zhengzhou 450000, P. R. China

^4^ Department of Physics, The Chinese University of Hong Kong, New Territories 999077, Hong Kong, P. R. China

^5^ Experimental Center of Advanced Materials School of Materials Science & Engineering, Beijing Institute of Technology, Beijing 100081, P. R. China

*Corresponding authors. E-mail: [chduan2025@henu.edu.cn](E:\\Nano-Micro Letters\\2026\\文章编校21761 21783-YJP\\文章编校21761 21783-YJP\\NML-2026-21761-YJP\\chduan2025@henu.edu.cn) (Chenghao Duan); glcai@ipe.ac.cn (Guilong Cai); sjzhang@ipe.ac.cn (Suojiang Zhang)

**S1 Experimental Section**

**S1.1 Other Characteristic**

The morphologies of thin films were obtained using a Nanoscope V atomic force microscope (AFM, Cypher ES, Oxford Instruments) in tapping mode. The absorption spectra measurements of thin films were performed on a UV-Vis spectrophotometer (UV-3600 PLUS, Shimadzu). The UPS of the perovskite films was achieved by a Shimadzu Kratos photoelectron spectrometer with a nonmonochromatic He Iα photon source (hν = 21.22 eV). Contact angles of water and diiodomethane on all films were measured using a contact angle system (Biolin Theta Lite, Biolin). X-ray diffraction (XRD) patterns were measured on Rigaku SmartLab SE. The SEM images were measured by using a JEOL JSM-7900F field-emission scanning electron microscope. The GIWAXS measurements were carried out with a Xeuss 2.0 SAXS/WAXS laboratory beamline using a Cu X-ray source (8.05 keV, 1.54 Å) and a Pilatus3R 300K detector. Liquid state proton (^1^H) nuclear magnetic resonance (NMR) measurements were recorded on a Bruker AscendTM 600. The FTIR spectroscopy was measured by a Fourier Transform Infrared Spectrometer (BRUKER INVENIO S). X-ray photoelectron spectroscopy (XPS) was measured on monochromatic Al-Ka (1486.6 eV) as the radiation source (Shimadzu, Axis Supra). The PL mapping spectra of perovskite films were obtained by a Laser Scanning Confocal Microscope (Enlitech, SPCM-1000). The TRPL measurements were carried out via the PicoQuant FluoQuant 300. The TRPL mapping spectra of perovskite films were measured by a laser scanning confocal microscope (SouthPort, JadeMAT). Time-of-flight secondary ion mass spectrometry (TOF-SIMS) measurements were conducted on a PHI nano TOF II (ULVAC PHI Inc., Japan).

**S1.2 DFT Characteristic**

We performed all density functional theory (DFT) calculations using the CP2K package [S1]. The Perdew-Burke-Ernzerhof (PBE) functional with Grimme D3 correction [S2], which incorporates a generalized-gradient approximation for the exchange-correlation functional, was employed for both the self-consistent field calculations and geometry optimization. The Goedecker-Teter-Hutter (GTH) pseudopotentials, DZVPMOLOPT-GTH basis sets were utilized to describe the molecules [S3-5]. A plane-wave energy cut-off of 500 Ry has been employed.

Interaction between the molecular layer and its neighboring layers: regarding SMAS/PVK and 2-FAS/PVK interface models, we investigated the interaction energy between molecules and the bottom surface of the perovskite. A two-layer (3*3) perovskite FAPbI_3_ (001) surface has been used for SMAS and 2-FAS adsorption. All adsorption calculations were done at the gamma point. The adsorption energy (Δ*E*ads) is defined as:

$$\Delta E_{ads}=E_{slab+molecule}-E_{slab}-E_{molecule}$$

Where *E*_slab_ and *E*_molecule_ are the dipole-corrected electronic energy of the slab and adsorbed molecule, respectively.

**S2 Supplementary Figures and Tables**

**Fig. S1** The molecular structures and ESP of **a** SMAS and **b** 2-FAS

**Fig. S2** SEM image and the elemental distribution of the FTO obtained by EDS characterization

**Fig. S3** SEM image and the elemental distribution of the SAM film obtained by EDS characterization

**Fig. S4** AFM image of the FTO substrate

**Fig. S5** Contact angles of the precursor solution with **a** SAM, **b** SAM/SMAS, and **c** SAM/2-FAS films. The water contact angle with **d** SAM, **e** SAM/SMAS, and **f** SAM/2-FAS films

**Fig. S6** C-AFM images of SAM, SAM/DMSO, SAM/SMAS, and SAM/2-FAS films at the nanometer scale

**Fig. S7** Optical transmission spectra of SAM, SAM/SMAS, and SAM/2-FAS film

**Fig. S8** UPS spectrum showing cutoff energy (left) for **a** SAM, **b** SAM/SMAS, and **c** SAM/2-FAS films. And cutoff energy (right) for **d** SAM, **e** SAM/SMAS, and **f** SAM/2-FAS firms

**Fig. S9** PL intensity during the anti-solvent spin-coating

**Fig. S10** Statistical distribution of grain size (bottom-view SEM) of **a** SAM, **b** SMAS, and **c** 2-FAS based perovskite films

**Fig. S11** The SEM cross-sectional view of **a** SAM, **b** SMAS, and **c** 2-FAS based perovskite films

**Fig. S12** **a** XRD patterns and **b** partial enlarged detail patterns of perovskite films based on SAM, SAM/SMAS, and SAM/2-FAS films

**Fig. S13** 2D GIWAXS pattern of **a** Control **b** SMAS **c** 2-FAS samples at an incident angle of 0.1°

**Fig. S14** GIXRD patterns with different instrumental tilt *ψ* values (the measured XRD patterns and fitted results with Gauss function) for perovskite films deposited on **a** SAM **b** SAM/SMAS and **c** SAM/2-FAS films.

**Fig. S15** Linear fits of perovskite films based on SAM, SAM/SMAS, and SAM/2-FAS films

**Fig. S16** **a** Absorption spectra and **b** Eg values of perovskite films deposited on SAM, SAM/SMAS, and SAM/2-FAS films

**Fig. S17** ^1^H nuclear magnetic resonance spectra of **a** SAM, SMAS, and SAM+SMAS, **b** SAM, 2-FAS, and SAM+2-FAS in DMSO-d6 solution

**Fig. S18 a** The full XPS spectra and **b** partial enlarged detail patterns of SAM, SMAS, and 2-FAS based perovskite films

**Fig. S19** XPS spectra of S 2*p* for **a** SMAS and **c** 2-FAS based perovskite films. XPS spectra of Na 1*s* for **b** SMAS and **d** 2-FAS based perovskite films

**Fig. S20** DFT-simulated conformations of Na^+^ from SMAS occupy the A position to form energy

**Fig. S21** DFT-simulated conformations of Na^+^ from 2-FAS occupy the A position to form energy

**Fig. S22** DFT-simulated conformations of the π–π function between 2-FAS and the SAMs layer

**Fig. S23** *J-V* curves of PSCs with different concentrations of **a** SMAS and **b** 2-FAS

**Fig. S24** The ESP of ortho-, meta-, and para-2-FAS (the arrow direction indicates the direction of the dipole moment)

**Fig. S25** *J-V* curves of the devices with ortho-, meta-, and para-2-FAS

**Fig. S26** *J-V* curves of the devices using SMAS or 2-FAS alone without a SAM layer

**Fig. S27** The statistics of **a** *V*_OC_, **b** FF, and **c** *J*_SC_ for devices based on SAM, SAM/SMAS and SAM/2-FAS firms

**Fig. S28** **a** Light soaking stability at nitrogen atmosphere based on unencapsulated-PSCs. **b** The thermal stability of the unencapsulated-PSCs under 65 **°**C heating in a nitrogen atmosphere. **c** Storage stability of the unencapsulated-PSCs under an air environment with 15-25% RH

**Fig. S29** MPP tracking stability of the unencapsulated devices under continuous one-sun illumination in a N_2_ atmosphere at room temperature

**Fig. S30** The EQE_EL_ plots of the control and 2-FAS devices

The electrical conductivity (*σ*) was calculated using the following Formula (S1):

$\sigma=\frac{L}{AR}\leftarrow$	(S1)

where L represents the thickness of the interface, A represents the effective area of the device, and R represents the resistance value.

The hole mobility (*μ*_hole_) was calculated using the following Formula (S2):

$\mu_{hole}=\frac{8JL^{3}}{9\varepsilon_{0}\varepsilon(V_{app}-V_{r}-V_{bi})^{2}}$	(S2)

where J is the electric current density, L represents the film thickness, V represents the voltage, ε_0_ and ε_r_ are the permittivity of vacuum and relative dielectric constant of the transport layer, respectively.

**Table S1** Calculated results for the electrical conductivity (*σ*) and hole mobility (*μ*_hole_) of pure HTL devices without and with SMAS/2-FAS

| Device | *σ* (S cm^-1^) | *μ*_hole_ (cm^2^ V^-1^ S^-1^) |
| --- | --- | --- |
| Control | 1.13× 10^-5^ | 1.41× 10^-3^ |
| SMAS | 3.47× 10^-5^ | 4.34× 10^-3^ |
| 2-FAS | 4.43× 10^-5^ | 5.54× 10^-3^ |

The work function (W_F_) of the film can be calculated by the following Formula (S3):

$W_{F}=hv-(E_{on set}-E_{cut off})$	*(*S3*)*

and the valence band (VBM) of thin film samples can be calculated by Formula (S4):

$$VBM=W_{F}-E_{on set}$$

**Table S2** Calculated parameters for the energy level of SAM, SAM/SMAS, and SAM/2-FAS firms

| Device | E_cutoff_ (eV) | E_onset_ (eV) | W_F_ (eV) | VBM (eV) |
| --- | --- | --- | --- | --- |
| Control | 16.36 | 0.43 | 4.86 | 5.29 |
| SMAS | 16.23 | 0.36 | 4.99 | 5.35 |
| 2-FAS | 16.16 | 0.33 | 5.06 | 5.39 |

**Table S3** PCE of PSCs with different concentrations of SMAS

| Device | *V*_OC_ (V) | *J*_SC_ (mA cm^-2^) | FF (%) | PCE (%) |
| --- | --- | --- | --- | --- |
| 0 mg/mL | 1.129  (1.127±0.002) | 25.83  (25.62±0.21) | 84.10  (83.34±0.76) | 24.58  (24.27±0.31) |
| 1.0mg/mL | 1.144  (1.143±0.001) | 26.07  (25.95±0.12) | 84.24  (83.43±0.81) | 25.13  (24.90±0.23) |
| 1.5mg/mL | 1.150  (1.148±0.002) | 25.63  (25.52±0.11) | 85.52  (84.90±0.62) | 25.22  (24.97±0.25) |
| 2.0mg/mL | 1.154  (1.153±0.001) | 25.82  (25.72±0.10) | 85.26  (84.68±0.58) | 25.41  (25.25±0.16) |
| 2.5 mg/mL | 1.134  (1.132±0.002) | 25.60  (25.48±0.12) | 84.59  (83.91±0.68) | 24.63  (24.40±0.23) |

**Table S4** PCE of PSCs with different concentrations of 2-FAS

| Device | *V*_OC_ (V) | *J*_SC_ (mA cm^-2^) | FF (%) | PCE (%) |
| --- | --- | --- | --- | --- |
| 0 mg/mL | 1.128  (1.125±0.03) | 25.82  (25.59±0.23) | 84.51  (83.71±0.80) | 24.61  (24.43±0.18) |
| 1.0mg/mL | 1.142  (1.140±0.02) | 26.15  (25.98±0.17) | 85.86  (85.13±0.73) | 25.66  (25.45±0.21) |
| 1.5mg/mL | 1.156  (1.155±0.01) | 26.12  (25.88±0.24) | 85.66  (85.01±0.65) | 25.86  (25.69±0.17) |
| 2.0mg/mL | 1.164  (1.163±0.01) | 26.13  (25.94±0.19) | 86.15  (85.61±0.54) | 26.21  (26.04±0.17) |
| 2.5 mg/mL | 1.141  (1.139±0.02) | 25.96  (25.70±0.26) | 85.23  (84.53±0.70) | 25.31  (25.09±0.22) |

**Table S5** PCE of the devices using SMAS or 2-FAS alone without a SAM layer

| Device | *V*_OC_ (V) | *J*_SC_ (mA cm^-2^) | FF (%) | PCE (%) |
| --- | --- | --- | --- | --- |
| SMAS | 0.822 | 23.93 | 79.90 | 15.57 |
| 2-FAS | 0.922 | 24.25 | 79.14 | 17.87 |
| SAM/SMAS | 1.154 | 25.82 | 85.26 | 25.41 |
| SAM/2-FAS | 1.164 | 26.13 | 86.15 | 26.21 |

**Table S6** Reported photovoltaic performance of the inverted PSCs with buried interfaces

| Perovskite constitution | Time | PCE (%) | Ref. |
| --- | --- | --- | --- |
| Cs_0.05_(FA_0.97_MA_0.03_)_0.95_Pb(I_0.97_Br_0.03_)_3_ | 2023 | 24.50 | [S6] |
| Cs_0.05_(FA_0.95_MA_0.05_)_0.95_Pb(I_0.95_Br_0.05_)_3_ | 2024 | 25.87 | [S7] |
| FA_0.85_MA_0.1_Cs_0.05_PbI_3_ | 2024 | 25.08 | [S8] |
| Cs_0.05_(FA_0.95_MA_0.05_)_0.95_PbI_3_ | 2024 | 25.92 | [S9] |
| FA_0.95_Cs_0.05_PbI_3_ | 2025 | 25.61 | [S10] |
| Cs_0.05_FA_0.85_MA_0.1_PbI_3_ | 2025 | 26.03 | [S11] |
| Cs_0.05_MA_0.1_FA_0.85_PbI_3_ | 2025 | 26.45 | [S12] |
| **Cs_0.05_FA_0.85_MA_0.1_PbI_3_** | **2025** | **26.21** | **This work** |

The open-circuit voltage (*V*_OC_) is fitted with the Formula (S5):

$V_{\mathrm{OC}}=\frac{nKTln(I)}{e}+constant$	(S5)

Where T is the absolute temperature, e represents the elementary charge, I represents the light intensity, and n indicates the ideality factor.

The defect density (N_trap_) in the perovskite is calculated by the following Formula (S6):

$N_{trap}=\frac{2\varepsilon_{0}\varepsilon V_{TFL}}{eL^{2}}$	(S6)

where V_TFL_ is the trap field limiting voltage, e is the unit electron charge, L is the thickness of the perovskite layer, ɛ_0_ denotes as the vacuum permittivity, and ɛ remarks as the relative permittivity of the perovskite.

The double exponential attenuation Formula (S7):

$f\left( x \right)=A_{1}exp(\frac{-t}{\tau_{1}})+A_{2}exp(\frac{-t}{\tau_{2}})$	(S7)

where $A_{1}$and $A_{2}$ represent relative amplitudes, $\tau_{1}$ and $\tau_{2}$indicate carrier lifetimes for fast and slow decay, respectively.

The average carrier lifetime ($\tau_{avg}$) can be calculated by the Formula (S8):

$\tau_{avg}=\frac{\sum{A_{i}\tau_{i}}^{2}}{\sum A_{i}\tau_{i}}$	(S8)

**Table S7** Carrier decay amplitude (A_1_, A_2_), times ($\tau_{1}$, $\tau_{2}$) of perovskite films based on SAM, SAM/SMAS, and SAM/2-FAS films

| Device | $\mathbf{A}_{\mathbf{1}}$ | $\mathbf{A}_{\mathbf{2}}$ | $\boldsymbol{\tau}_{\mathbf{1}}$(ns) | $\boldsymbol{\tau}_{\mathbf{2}}$(ns) | $\boldsymbol{\tau}_{\boldsymbol{avg}}$(ns) |
| --- | --- | --- | --- | --- | --- |
| Control | 2516.02 | 2419.02 | 10.89 | 193.58 | 183.48 |
| SMAS | 2915.44 | 2303.12 | 8.12 | 116.22 | 107.44 |
| 2-FAS | 3386.42 | 2110.26 | 4.77 | 62.60 | 56.30 |

The non-radiative recombination energy loss (Δ*V*_OC, nonrad_) of the device was estimated from the EQE_EL_ value measured under maximum applied bias (close to *V*_OC_), according to the following Formula (S9):

${\Delta V}_{non-rad}=\frac{k_{B}T}{q}ln(\frac{1}{{EQE}_{EL}})$	(S9)

**Supplementary References**

[S1] J. Hutter, M. Iannuzzi, F. Schiffmann, J. VandeVondele, CP2k: Atomistic simulations of condensed matter systems. Wiley Interdisciplinary Reviews-Computational Molecular Science. **4**(1), 15-25 (2014). <https://doi.org/10.1002/wcms.1159>
[S2] S. Grimme, Semiempirical GGA-type density functional constructed with a long-range dispersion correction. J. Compu. Chem. **27**(15), 1787-1799 (2006). <https://doi.org/10.1002/jcc.20495>
[S3] J. VandeVondele, J. Hutter, Gaussian basis sets for accurate calculations on molecular systems in gas and condensed phases. J. Chem. Phys. **127**(11), 114105 (2007). <https://doi.org/10.1063/1.2770708>
[S4] S. Goedecker, M. Teter, J. Hutter, Separable dual-space Gaussian pseudopotentials. Phys. Rev. B. **54**(3), 1703-1710 (1996). <https://doi.org/10.1103/PhysRevB.54.1703>
[S5] C. Hartwigsen, S. Goedecker, J. Hutter, Relativistic separable dual-space Gaussian pseudopotentials from H to Rn. Phys. Rev. B. **58**(7), 3641-3662 (1998). <https://doi.org/10.1103/PhysRevB.58.3641>
[S6] B. Niu, H. Liu, Y. Huang, E. Gu, M. Yan et al., Multifunctional hybrid interfacial layers for high‐performance inverted perovskite solar cells. Adv. Mater. **35**(21), 212258 (2023). <https://doi.org/10.1002/adma.202212258>
[S7] X. He, H. Chen, J. Yang, T. Wang, X. Pu et al., Enhancing hole transport uniformity for efficient inverted perovskite solar cells through optimizing buried interface contacts and suppressing interface recombination. Angew. Chem. Int. Ed. **63**(52), e202412601 (2024). https://doi.org/10.1002/anie.202412601
[S8] W. Zhang, J. Chen, C. Guan, T. Qiu, X. Shi et al., Harnessing dual hydrogen bonding and lewis acid–base interactions for bio-inspired symmetry-breaking electrolytes in aqueous zinc‐ion batteries. Angew. Chem. Int. Ed. **64**(43), e202516282 (2025). <https://doi.org/10.1002/anie.202516282>
[S9] X. Chen, Q. Wang, H. Wei, J. W. Yang, Y. Q. Yao et al., Minimizing the buried interfacial energy loss using a fluorine-substituted small molecule for 25.92%-efficiency and stable inverted perovskite solar cells. Energy Environ Sci. **17**(19), 7342-7354 (2024). <https://doi.org/10.1039/d4ee02964j>
[S10] L. Deng, P. Zhao, L. X. Ren, X. Zhang, Z. Y. Li et al., Bifacial defect management of buried interface for enhanced photovoltaic performance of inverted-perovskite devices with precise regulation of charge transport. Small Methods. **9**(9), e01052 (2025). <https://doi.org/10.1002/smtd.202501052>
[S11] Z. L. Song, Y. Zou, Y. P. Gao, X. B. Gao, et al., Buried and bulk synergistic engineering enables high-performance inverted 2D/3D perovskite solar cells. Energy Environ Sci. **18**(8), 3740-3749 (2025). <https://doi.org/10.1039/d5ee00156k>
[S12] Z. Zhou, R. Yang, B. Zhang, Z. Zhuang, J. Wang et al., Molecular bridge regulation of buried interface in perovskite solar cells. Adv. Mater. **38**(17), e19267 (2026). <https://doi.org/10.1002/adma.202519267>
